# Electrical and photocatalytic properties of boron-doped ZnO nanostructure grown on PET–ITO flexible substrates by hydrothermal method

**DOI:** 10.1038/srep42615

**Published:** 2017-02-13

**Authors:** Wei Wang, Taotao Ai, Qi Yu

**Affiliations:** 1 School of Materials Science and Engineering, Shaanxi Sci-Tech University, Shaanxi Hanzhong 723000, P.R. China

## Abstract

Boron-doped zinc oxide sheet-spheres were synthesized on PET**–**ITO flexible substrates using a hydrothermal method at 90 °C for 5 h. The results of X-ray diffraction and X-ray photoelectron spectroscopy indicated that the B atoms were successfully doped into the ZnO lattice, the incorporation of B led to an increase in the lattice constant of ZnO and a change in its internal stress. The growth mechanism of pure ZnO nanorods and B-doped ZnO sheet-spheres was specifically investigated. The as-prepared BZO/PET**–**ITO heterojunction possessed obvious rectification properties and its positive turn-on voltage was 0.4 V. The carrier transport mechanisms involved three models such as hot carrier tunneling theory, tunneling recombination, and series-resistance effect were explored. The BZO/PET**–**ITO nanostructures were more effective than pure ZnO to degrade the RY 15, and the degradation rate reached 41.45%. The decomposition process with BZO nanostructure followed first-order reaction kinetics. The photocurrent and electrochemical impedance spectroscopy revealed that the B-doping could promote the separation of photo-generated electron-hole pairs, which was beneficial to enhance the photocatalytic activity. The photocurrent density of B-doped and pure ZnO/PET**–**ITO were 0.055 mA/cm^2^ and 0.016 mA/cm^2^, respectively. The photocatalytic mechanism of the sample was analyzed by the energy band theory.

Zinc oxide (ZnO), as a direct wide band gap II-VI semiconductor and a prominent member of the family of transparent conductive oxide (TCO) films, has been extensively investigated, which possesses excellent characteristics with a band gap of 3.37 eV at room temperature^1^, high optical transmittance, and outstanding short-wavelength luminescence. Also, the thermal ionization energy of ZnO is 26 meV, and its exciton binding energy (60 MeV)^2^ is more than twice that of GaN (25 MeV), so it can realize efficient exciton-stimulated emission at room temperature, and has a diverse range of applications in devices such as blue-violet detectors, thin-film solar cells, and transparent thin film transistors^3^,^4^,^5^. Intrinsic ZnO, belongs to a polar semiconductor, presents n-type conductivity and contains many intrinsic defects. At present, the electrical and optical performances of ZnO can be greatly improved by doping impurity elements into the crystal lattice^6^,^7^,^8^, which contributes to increase the electron concentration in the conduction band of ZnO, as well as improves its transparency and stability in the visible-near-infrared region. Abduev *et al*.^8^ detailedly investigated the effect of B-doping on the electrical properties of Ga-doped ZnO thin films, and found that the resistivity of B-doped GZO thin films decreased contrast to the sample of the un-doped boron. Tsay *et al*.^9^ reported the influence of B-doping concentration on the electrical and optical properties of ZnO thin films, in which the Hall mobility and electron concentration of the 1% B-doped ZnO thin film achieved 17.9 cm^2^/V·s and 1.2 × 10^15^ cm^−3^, respectively. The potential applications of B-doped ZnO (BZO) for using in diluted magnetic semiconductors and the transparent electrode of thin-film solar cells^7^ have been widely concerned. ZnO is also a photocatalytic material, which can generate a photoelectron-hole pair under ultraviolet (UV) light at wavelengths below 387 nm and shows a good photocatalytic degradation in an acid or alkaline medium. Therefore, ZnO displays great promise as a high-activity photocatalyst^10^,^11^,^12^.

At present, most of the practical TCO films are usually prepared on a hard substrate, e.g., a glass substrate^13^ or a silicon wafer^14^. A conductive film prepared on a flexible substrate can not only retain the photoelectric properties of the deposited film, but also has advantages such as high flexion, low weight, and durability. Polyethylene terephthalate (PET) is a semi-crystalline thermoplastic polymer possessing both high transmittance in the visible light range and outstanding heat resistance (200 °C) relative to other polymers, so it can be used as an effective flexible substrate material^15^. However, due to the rough surface of PET, and larger thermal expansion coefficient than ZnO, greatly hinder the growth of ZnO on PET. ITO (tin-doped indium oxide) film not only has a high transmittance and conductivity, but also can perform the function of a buffer layer to prevent the infiltration of water vapor and oxygen, as well as reduce the residual stress and structural defects in the film. Furthermore, ITO layer as a photo-generated electron collecting layer can enhance photocatalytic activity, and affect electron transport the same as MgO buffer layer^16^,^17^. However, the properties of ITO films are unstable when it is immersed in a wide range of aggressive aqueous solutions and its performance are easily affected by the pH of the solution^18^. Besides, ITO-coated PET substrate requires low temperature processing. Therefore, it is necessary to adjust the growth parameters reasonably during the experiment process, to meet the needs of the growth of B-ZnO or pure ZnO nanomaterials and keep the properties stability of PET**–**ITO substrate. Thus, it is desired to obtain flexible electronic and optoelectronic devices with excellent properties based on a PET**–**ITO substrate.

In this study, sheet-sphere-shaped BZO were prepared by a hydrothermal technique on a PET**–**ITO flexible substrate at low temperature (90 °C). The effect of B-doping on the morphology and crystal growth of ZnO and the formation mechanisms of ZnO nanostructures were discussed. We tested current**–**voltage (I**–**V) characteristics of the constructed BZO/PET**–**ITO heterojunction in order to analyze system carrier transport mechanisms and further studied the electrical performance. Photocatalytic degradation experiment was carried out with a solution of reactive yellow 15 (RY 15, a non-ferrous azo dye). Finally, the light-catalyzed reaction process of ZnO nanostructures was studied based on the energy band theory.

## Methods

### Specimen synthesis

All reagents used in the experimental process were analytically pure. In order to hydrothermally grow ZnO nanostructures, zinc nitrate hexahydrate (Zn(NO_3_)_2_·6H_2_O) and hexamethylenetetramine (HMT, C_6_H_12_N_4_) were exactly weighed using a analytical balance with an accuracy of 10^−4^ g, and were dissolved in deionized water, producing precursor solution of 40 mL at a concentration of 0.05 M. Boric acid (H_3_BO_3_), at a concentration of 0.15 M, which provided the source of boron, was mixed with the as-prepared solution and stirred with a magnetic mixer for 30 min. Next, using a small ion-sputtering apparatus, a thin seed layer of ZnO was sputtered onto the surface of the as-treated PET**–**ITO substrate with the size of 2 cm × 1 cm to reduce the lattice mismatch between ZnO and the substrate. Among that, the surface resistivity of PET**–**ITO flexible substrate is 30 Ω/sq^19^, which is lower than that of the pure PET (60 Ω/sq^20^), but higher than that of ITO substrate (14 Ω/sq^20^). Process parameters were as follows: sputtering time, 5 min; sputtering current, 6–8 mA; vacuum pressure, 0.1 mbar. With that, the PET**–**ITO coated with the seed layer was dipped into the as-obtained aqueous solution and sealed by means of plastic wrap. Finally, the device was transferred to an electric oven and heated at a constant temperature of 90 °C for 5 h, so as to develop the BZO nanostructure. After cooling to room temperature (defined as 20–25 °C), the product was gradually washed with distilled water and then dried naturally in air.

### Characterization of the products

The morphology of the prepared samples was observed using scanning electron microscopy (SEM) (JEOL, JSM-6700F) with an accelerating voltage of 20 kV. The phase composition was analyzed by X-ray diffraction (XRD) with Cu-Kα radiation (Rigaku, D/Max 2200 PC). X-ray photoelectron spectroscopy (XPS) measurements were performed on an ESCALAB 250Xi spectrometer (excitation source of Al-Kα) to study the composition and chemical state of the sample surface. The I**–**V characteristics of the heterojunction were measured using a digital source meter (Keithley, 2400) with an accuracy of 10^−12^ A, and the photocatalytic activity of ZnO was evaluated by the degradation and decolorization of the RY 15 solution under UV light irradiation. The photochemical characteristics of the photocatalysts were examined at the electrochemical workstation of CHI660E, and the photocurrent and electrochemical impedance spectroscopy (EIS) test were implemented in three-electrode working mode.

### Photocatalytic degradation experiment

At room temperature, the RY 15 solution (10 mg/L) of 4 mL was added into a 20 cm-long quartz tube. The sample of BZO/PET**–**ITO was suspended below two-thirds of the liquid level by fine copper wire, and paralleled with the mercury lamp, in order to increase the specimen light irradiation area. A high-pressure mercury lamp with a primary wavelength of 365 nm at a power of 500 W was placed 1 m from the sample, providing the UV source. Throughout the reaction process, magnetic stirring was used to enhance the mass transfer, with sampling (2 mL) performed once every 20 min. In order to discuss the photocatalytic activities of ZnO, the concentration of RY 15 was analyzed with the variation of reaction time by the direct colorimetric method at a wavelength of 414.5 nm. The degradation rate of RY 15 was calculated by the following formula:


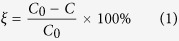


In equation (1), ξ represents the degradation rate, *C*_*0*_ and *C* represent the initial concentration of RY 15 solution and the concentration after photocatalytic degradation, respectively.

## Results and Discussion

### Analysis of phase composition

**Figure 1:**
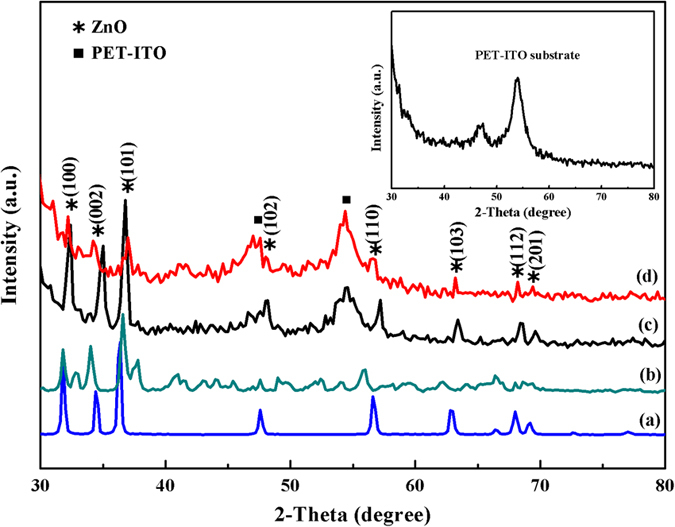
XRD patterns of pure ZnO (**a**), B-doped ZnO (**b**), ZnO/PET**–**ITO (**c**) and BZO/PET**–**ITO (**d**). The inset is the X-ray diffraction peak of the PET**–**ITO substrate alone.

Figure 1 shows the XRD patterns of pure and B-doped ZnO powders prepared by hydrothermal method and ZnO nanomaterials with two different morphologies grown on the PET**–**ITO substrate. By comparing the XRD peaks in the illustration, the marked diffraction peaks are all characteristic peaks of ZnO, except for the two diffraction peaks at 47° and 54.6° that are attributed to the PET**–**ITO substrate. The peak positions of the pure and B-doped ZnO samples are in conformity with the JCPDS card standard (No. 89–1397), verifying the hexagonal wurtzite structure of the products. Differences in Fig. 1 between the diffraction peaks show that the feature peak of the BZO nanostructure slightly shifts toward the low-angle direction comparing to that of the un-doped ZnO. However, the peak of ZnO grown on the PET**–**ITO substrate moves in a large angle direction in contrast to the ZnO powder without any substrate. Therefore, it can be demonstrated that the B-doping and the PET**–**ITO substrate have important effect on the internal stress of ZnO, which further gives rise to lattice distortion. The diffraction intensity of Fig. 1d is obviously smaller than that of Fig. 1c, and it is speculated that the B-doping deteriorated the crystallinity of ZnO to a certain extent. Moreover, there are no boron compounds and other impurities found in the specimen of BZO, indicating the purity of the as-fabricated samples is higher.

The lattice constants were calculated using the Bragg equation (2) and the interplanar spacing formula for a hexagonal system (3):


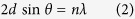



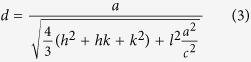


Here, *d* is the interplanar spacing, *θ* is diffraction angle, *n* is the diffraction series, and (*hkl*) is the Miller indices of the crystal face. After calculating, we found that the lattice constants (*a* = 3.206 Å, *c* = 5.233 Å) and the crystal plane spacing of BZO/PET**–**ITO are both larger than that of ZnO/PET**–**ITO (*a* = 3.196 Å, *c* = 5.134 Å). It is generally believed that there are three potential mechanisms about B atoms doped into the ZnO crystal lattice^21^. One is to occupy the octahedral interstice. This is due to the fact that in the crystal structure of ZnO, O^2−^ ions are arranged in accordance with the hexagonal close-packed, Zn^2+^ ions are interspersed into a tetrahedral interstice formed by half of the O^2−^ ions, while the octahedral interstice is produced by six O^2−^ ions or six Zn^2+^ ions in close packing. The eight faces of the octahedral interstice are triangles, consisting of six sides and two bottoms rotated 60° relative to each other. Since all of the octahedral voids are empty, in n-type ZnO with higher Fermi levels, as-generated octahedral interstices are most likely to be occupied^22^. In addition, the remaining two mechanisms are O or Zn atoms replaced by B, respectively. There is no doubt that the radius of B^3+^ (0.02 nm) is smaller than that of O^2−^ (0.14 nm) and Zn^2+^ (0.074 nm). If the O or Zn atoms are replaced by B, the crystal plane spacing will shrink, and result in the diffraction peaks shifting to higher angle, which is inconsistent with the experimental results according to Bragg’s equation. Consequently, the B atoms are likely to occupy in the octahedral interstices.

**Figure 2:**
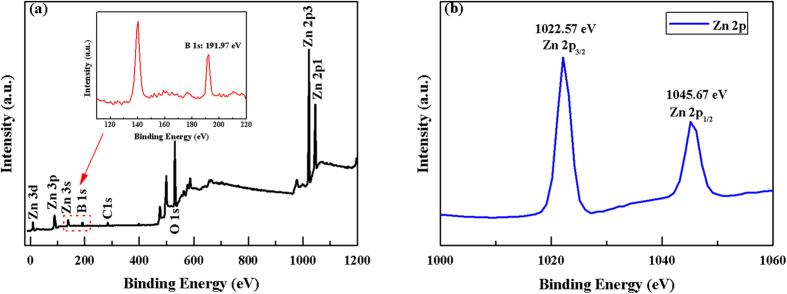
XPS survey spectra of the BZO/PET**–**ITO nanostructure (**a**) and high resolution spectrum of Zn 2p (**b**). The illustration in (**a**) corresponds to the boron 1s peak.

The XPS survey spectrum of the B-doped ZnO sheet-spheres indicates the existence of the Zn, O, and B elements, as shown clearly in Fig. 2a. In addition, the weaker C 1 s peak at 284.87 eV is also detected, which might be caused by the fact that the sample surface adsorbed very little CO_2_ in the air^23^. The binding energy peak of B 1 s shown in the inset of Fig. 2a is located at 191.97 eV, meaning that the doping B atoms are in the trivalent state (B^3+^)^24^. The atomic concentration of B in ZnO is calculated to be 8.69 at.% using element peak area and sensitivity factor. The peak at 531.27 eV corresponds to the binding energy of O 1s, may be ascribed to the characteristic peak of O^2−^ in wurtzite ZnO^25^. The corresponding high-resolution spectrum of Zn 2p peaks are exhibited in Fig. 2b. As the electrons in the atom have booth orbital and spin motions, the 2p energy level splitting under the action of the spin-orbit coupling, result in the energy levels generating double peaks on the XPS spectra. The two peaks locating to 1022.57 eV and 1045.67 eV in Fig. 2b are assigned to Zn 2p3/2 and Zn 2p1/2, respectively, revealing that most of the Zn in the B-ZnO system exist in the form of the Zn^2+^ ions^26^.

### Microstructure and growth habit

**Figure 3:**
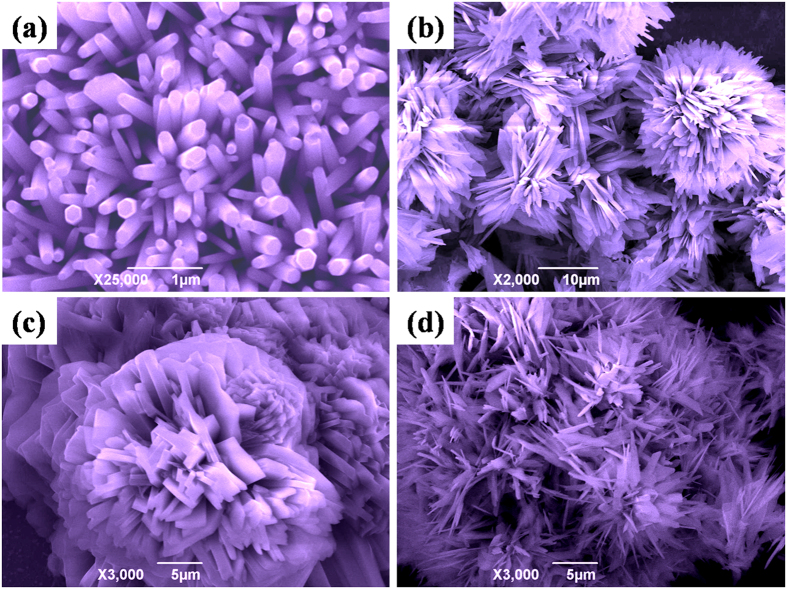
SEM images of (**a**) un-doped ZnO nanorods, (**b**) 15% B-doped ZnO sheet-spheres, (**c**) 10% BZO and (**d**) 20% BZO fabricated by the hydrothermal method (90 °C, 5 h).

The SEM images of pure and 15% B-doped ZnO/PET**–**ITO are shown in Fig. 3a and b. It can be seen from Fig. 3a that the ZnO nanorods obtained possess a smooth surface and a hexagonal cross section with diameters of 83–250 nm, and with a high-density distribution on the substrate. The ZnO nanorods are arranged irregularly and not all grow vertically on the substrate. In other words, the polar (002) face of the hexagonal system does not provide the strongest diffraction peak. As shown in Fig. 3b, sheet-spheres ZnO are obtained and arranged tightly. The specific spheres with a diameter of 12.5 μm cover with a mass of ZnO sheets interconnecting each other with a thickness of 0.667 μm. In order to roughly investigate the effect of boron content on the morphological evolution of ZnO, SEM images of the samples doping with 10% and 20% H_3_BO_3_ solution are presented in Fig. 3c and d. In Fig. 3c, sheet-spherical structure can be obtained, but the size of the sheets on the spheres are not uniform and very thickness, which is not as good as that of the product in Fig. 3b. When the doping amount is increased to 20% (Fig. 3d), near spinal structure is presented, which is different from sphere structure. From the above analysis, we can infer that the addition of H_3_BO_3_ solution vastly affects the growth habit of ZnO crystal, which leads to varied morphologies.

The basic chemical reactions in the course of hydrothermally synthesizing ZnO nanostructures are as follows:


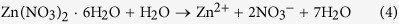



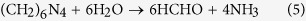



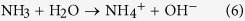



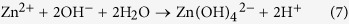



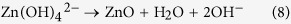


HMT acted as a surface active agent, decomposed into formaldehyde and ammonia, which provided the hydroxyl ions (OH^−^) combined with the central cationic Zn^2+^ to create stable Zn(OH)_4_^2−^ ions. These ions were continuously transported to the surface of the substrate. Zn(OH)_4_^2−^ growth units were easily adsorbed on the substrate by the induction of a seed layer on the PET**–**ITO surface, and they produced the ZnO after a series of repeated dehydration reactions. The heterogeneous nucleation began once the concentration of ZnO in the mixed solution reached the irreducible minimum of saturation corresponding to the critical free energy. Homogeneous nucleation occurred difficultly, because it’s necessary to overcome the upper activation energy barrier. Once nucleation did occur, the growth process of ZnO would break out simultaneously. The morphology of ZnO crystal is closely related to the crystalline behavior under hydrothermal conditions. Wurtzite ZnO as a polar crystal has an asymmetric structure and two polar surfaces; a positively-charged Zn polar surface (0001) and an electronegative O plane (000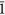). The (0001) crystal face has the lowest surface free energy, and its growth rate is the largest of all the crystal surfaces, as follows: V(0001) > V(10
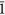
1) > V(10
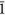
0) > V(000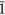)^27^. Zn(OH)_4_^2−^ growth units with a negative charge have a dipole characteristic, which preferentially aggregates on the positive surface of ZnO. As a result, ZnO crystals grow rapidly along the (0001) direction and gradually form the ZnO nanorods with a hexagonal cross profile.

**Figure 4:**
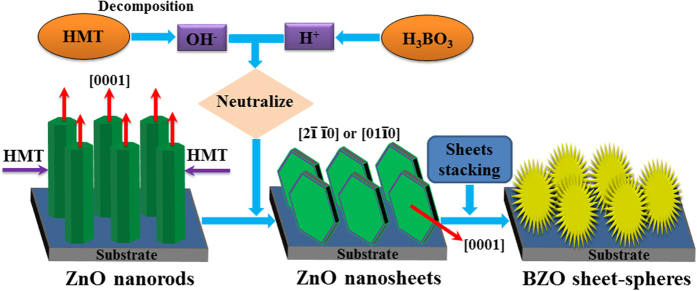
The growth mechanism of sheet-sphere-shaped BZO nanostructure.

The schematic diagram of the formation mechanism of lamellar spherical B-doped ZnO is shown in Fig. 4. HMT not only supplies the configurational OH^−^ ions, but also can be used as an organic coating agent, attached to the non-polarized plane of ZnO to inhibit the growth of the ZnO crystal nuclei during the process. When H_3_BO_3_ was added in, H^+^ was introduced into the precursor solution and neutralized with the existing OH^−^, resulted in the HMT further decomposed, and decreased the shielding effect of the non-polar surface and increased the growth rate. Various nanosheets appeared, which could grow along the directions of [00
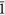
0] and [0001] or [2
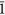

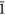
0] and [01
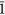
0]^28^. With the addition of H^+^, there are two influences: (i) The aqueous solution transforms from alkaline to neutral, so the solubility of ZnO will be lower, result in the supersaturation of the whole solution increased; (ii) The chemical reaction (5) will be forced to proceed. It leads to second nucleating and the formation of lamellar structures, and every two lamellar structures are connected with each other. There is an angle between the two nanosheets which may be affected by the van der Waals forces. As the surface tension and energy of the spherical structure is the lowest, the spherical ZnO composed of numerous nanosheets are obtained so as to reduce the total interface energy of the system.

### Electrical properties

**Figure 5:**
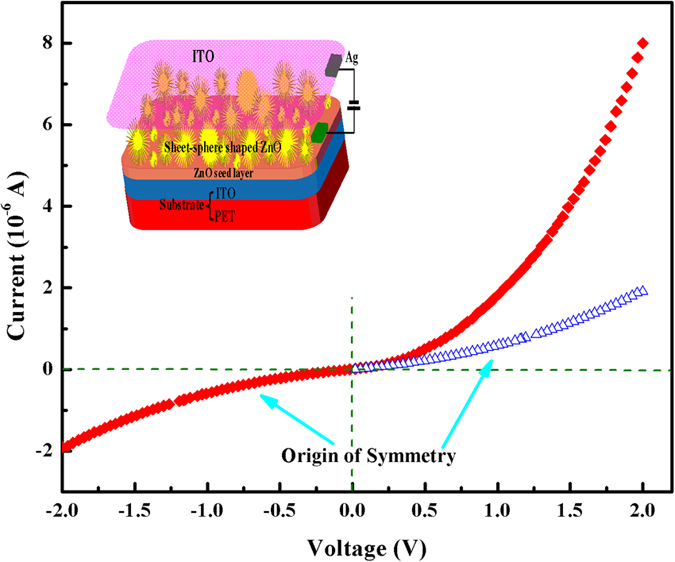
I–V characteristic of the BZO/PET–ITO heterojunction. The inset is a schematic diagram of equipment structure.

The I–V characteristics of BZO sheet-spheres grown on p-type PET–ITO are depicted in Fig. 5. The inset is a schematic diagram of the BZO/PET–ITO heterojunction diode. The PET–ITO substrate and transparent conductive ITO glass pressed on the top surface of the sheet-sphere-shaped ZnO were used as the anode and the cathode, respectively. The copper conductor was connected to the two electrodes of the diode by conductive silver paste, thus operating as Schottky contacts^29^ to assemble into a completely closed circuit. As seen in Fig. 5, the asymmetry of the I–V curve shows that the diode has certain rectifying performance, and its positive turn-on voltage is 0.4 V. When the applied forward bias voltage reaches 2 V, the current (I_F_) reaches 8 μA; the reverse leakage current (I_R_) reaches −1.9 μA at −2 V, result in a rectification ratio (I_F_/I_R_) of 4.2. In the negative direction, the reverse current is observed to be in an unsaturated state and its value is relatively large, which demonstrates that the rectifying characteristic of the prepared heterojunction is not particularly satisfactory.

**Figure 6:**
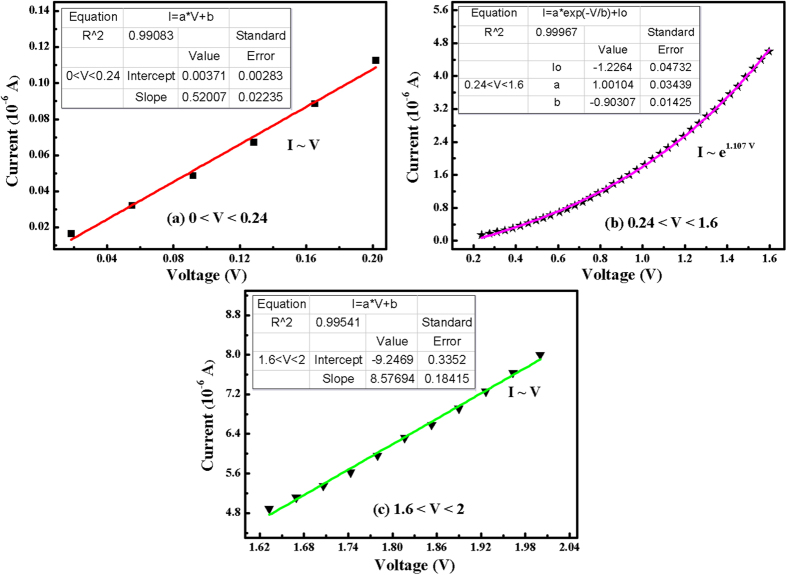
The fitting of I–V curve of the heterojunction diode with a forward bias.

When the BZO/PET–ITO heterojunction is under the forward bias voltage, the applied voltage chiefly acts on the barrier region with very small carrier concentration and higher resistance, so that the electric field in the space charge region and drift motion are weakened, and the original balance between the diffusive movement and drift motion of the carrier is also destroyed. Consequently, the diffusion flow is larger than the drift current. Electrons (holes) pass through the barrier region into the p- (n-) region, to initiate the accumulation of electrons (holes) and a concentration gradient at the boundary of the region. As a result, the electrons (holes) reflect as non-equilibrium minority carriers of p- (n-) region, diffuse from the boundary of the p- (n-) region to the inner region and form an electron (hole) diffusion current. It is justly because of the non-equilibrium minority carrier’s electrical injection theory that leads to the generation of forward current. The fitting of the I–V curve of the BZO/PET–ITO heterojunction diode at forward bias is described in Fig. 6, which presents three diverse fitting equations with different forward voltage area. In Fig. 6a, the relational expression between current and voltage (I = 0.52 V + 0.0037) is calculated in the range of 0 < V < 0.24, which can be regarded as obeying Ohm’s Law (I ∝ V). It is due to the concentration of electric injected minority carriers initially being relatively small, lower than that of the hot carrier; meanwhile, the hot carrier tunneling mechanism plays a leading role in the current transport principle. When the bias voltage increases to the range of 0.24 < V < 1.6, the I–V equation follows an exponential path (I ∝ e^1.107 V^), as shown in Fig. 6b. Two materials with different lattice constants are closely combined together to form a heterojunction, which gives rise to the issue of lattice mismatch and the introduction of multiple interface states at the contact surface. Thus, the relation under the circumstances can be explained by the tunnel recombination model^30^. For the third area (1.6 < V < 2), the I–V equation follows a linear relationship (I ∝ V) again, but the slope (8.58) is much larger than that of the fitted regression line in Fig. 6a, and this relation is possibly attributed to the series resistance effect. When the applied positive voltage is larger, the as-formed Schottky contact will assume a part of the voltage drop, which cause the following conclusions: Forward bias is composed of three sections, characterized by the voltage drop of the diffusion region V_1_, the potential drop of the barrier region V_2_, and a resistance voltage drop V_3_. Therefore, compared to the exponential relationship (Fig. 6b), the value of V_2_ will be reduced and the corresponding forward current is slowly increased to approach linear growth.

When the backward voltage is applied, the electric field in the barrier region is enhanced due to the electric field direction generated by the reverse voltage in the barrier region which consistent with a built-in electric field. Therefore, the drift motion is strengthened, as a result, the drift flux is larger than the diffusion flow, further causes the hole (electron) in the boundary of the n- (p-) region to be driven to the p- (n-) region by the strong electric field in the barrier region to form a hole-diffusion current (electron diffusion current) under reverse bias. The minority carrier extraction leads to the reverse current. The reverse leakage current is unsaturated in Fig. 5, which may arise from the following two reasons: (i) Since the band gap of ZnO is relatively wide and its intrinsic carrier concentration is small, the value of the net electricity generated in the barrier region is much larger than that of the reverse diffusion current, demonstrate that the net current of the barrier region plays a dominate role in the reverse current. Due to the width of barrier zone is broadened with increasing reverse bias voltage, the net current of barrier region will be unsaturated and slowly increased. Consequently, leading to an unsaturated state of the countercurrent during the whole process of reverse-loading voltage. (ii) There are many defects at the interface, which can create the defect auxiliary leakage current^31^.

### Photocatalytic performance

**Figure 7:**
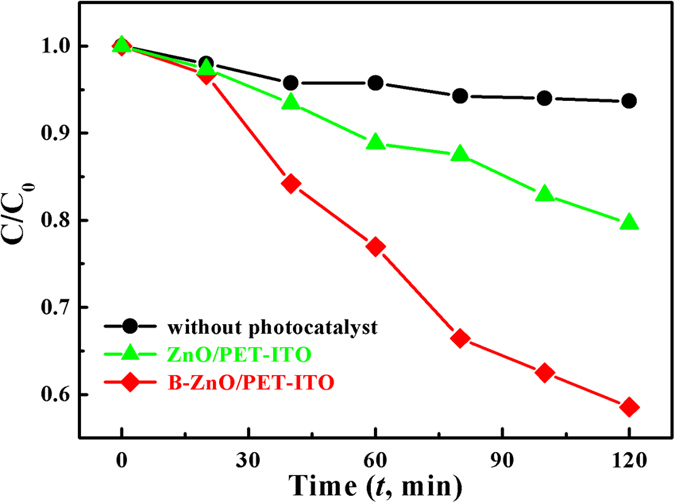
Photocatalytic degradation curve of undoped ZnO/PET–ITO, B-doped ZnO/PET–ITO, and without photocatalyst.

Figure 7 shows the evolution of the photocatalytic degradation of RY 15 over time. After UV irradiation for 120 min, the degradation of RY 15 solution without any photocatalysts is negligible. The degradation rate of the BZO/PET–ITO heterojunction as a photocatalyst is 41.45%, which is obviously higher than that of the un-doped ZnO nanorods (20.4%) under the same irradiation conditions. It reveals that ZnO/PET–ITO has excellent photocatalytic properties, and can be used as a catalyst to degrade the non-ferrous azo dye. Furthermore, the photocatalytic activity can be expressly improved with the doping of boron atoms in the nanostructure of ZnO.

**Figure 8:**
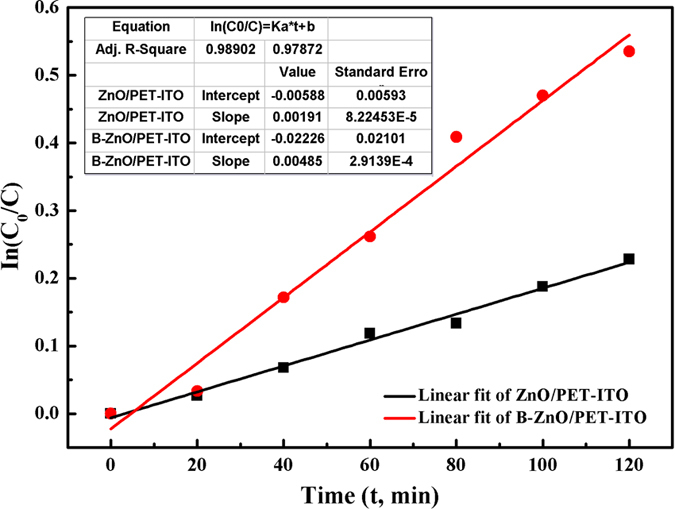
Kinetics fitting of In(C_0_/C) and decomposition time (t) with respect to pure and B-doped ZnO/PET–ITO degraded of RY 15.

The scatter diagrams of In(C_0_/C) over time along with the corresponding linear fittings are shown in Fig. 8. A good linear relationship between In(C_0_/C) and the time of the pure and B-doped ZnO/PET–ITO samples are examined, where the corresponding correlation coefficients (R^2^) determine as 0.989 and 0.979, respectively, both of which are close to 1. It demonstrates that the decomposition process with ZnO nanostructures follows first-order reaction kinetics (In(C_0_/C) = K_a_t + b, where K_a_ is the reaction rate constant). The kinetic equation and rate constant in the process of degrading the RY 15 solution with pure or B-doped ZnO/PET–ITO can be obtained by calculation, as exhibited in Table 1 below. The photocatalytic activity can be estimated by the kinetic rate constants of RY 15 degradation. The results of Table 1 show that the photocatalytic degradation capacity of BZO/PET–ITO is stronger than ZnO/PET–ITO, although the kinetic constants of BZO is lower than that of SiO_2_/ZnO nanoparticles (0.026 min^−1^)^32^. Some researches showed that the crystalline morphology had a profound effect on the photocatalytic performance of ZnO^12^,^33^,^34^. Therefore, from the perspective of the crystalline structure, the morphology of ZnO is changed after the incorporation of boron into the sample to produce the lamellar spherical ZnO, which is easily generated many defects at the bonding points of the two sheets. This defects provide more reactive centers for the catalytic process, thus the rate of photocatalytic degradation is enhanced.

**Figure 9:**
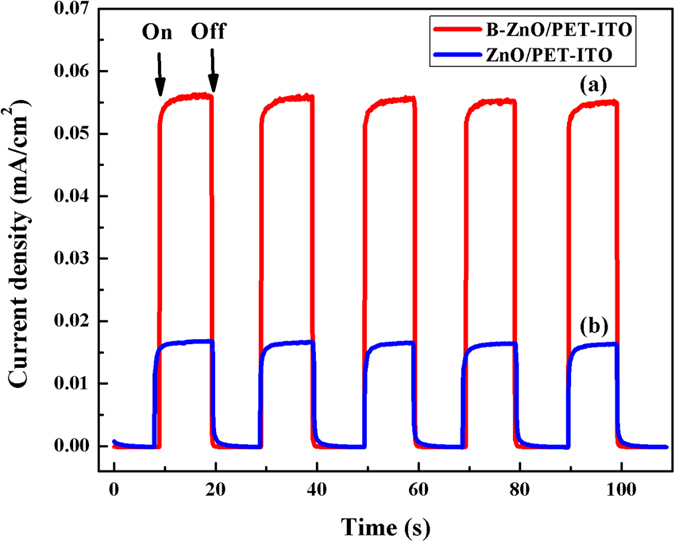
Photocurrent transient response spectra of B-doped (**a**) and pure (**b**) ZnO/PET-ITO under UV illumination.

In order to analyze the effect of B-doping on the recombination probability of photo-generated carriers in the sample, the photocurrent transient response of 15% B doped and pure ZnO/PET-ITO under UV irradiation are measured, as shown in Fig. 9. After several times of on-off intermittent irradiation, the current densities of the two different samples are basically constant, which demonstrates that the stability of the samples acting as electrodes is outstanding and the photocurrent is reversible. The photocurrent density of B-ZnO is obviously higher than that of pure ZnO, indicating that the photoelectric conversion efficiency of ZnO after B-doping is improved. The photocurrent density of BZO/PET-ITO is 0.055 mA/cm^2^, which is about 3.4 times that of pure ZnO/PET-ITO (0.016 mA/cm^2^). The enhancement of the photocurrent density of BZO/PET-ITO reveals that the separation efficiency of photo-generated electron-hole pairs in the sample are promoted after B-doping, result in the decrease of its recombination probability.

**Figure 10:**
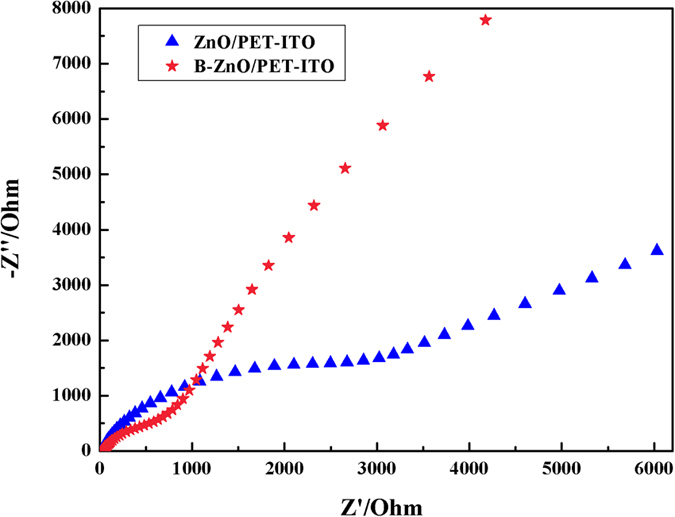
Electrochemical impedance spectroscopy of ZnO/PET-ITO and BZO/PET-ITO.

The Nyquist type electrochemical impedance spectroscopy of pure and B-doped ZnO/PET-ITO are described in Fig. 10, which can be further verified that the doping of B can effectively promote the separation of electron-hole pairs. The radii of the arcs in the EIS reflect the magnitude of the interfacial layer resistance produced by the electrode surface, and the interfacial charge carrier’s separation efficiency for the photocatalyst can be judged visually. It can be seen in Fig. 10 that the arc radius of the impedance curve belonging to the BZO/PET-ITO sample is significantly smaller than that of ZnO/PET-ITO, which displays that its interfacial layer resistance and surface charge transfer are distinctly decreased. Therefore, the recombination of charge carrier is suppressed by the incorporation of boron, and the photocatalytic efficiency is improved. The results are in agreement with that of the transient response spectra of photocurrent.

**Figure 11:**
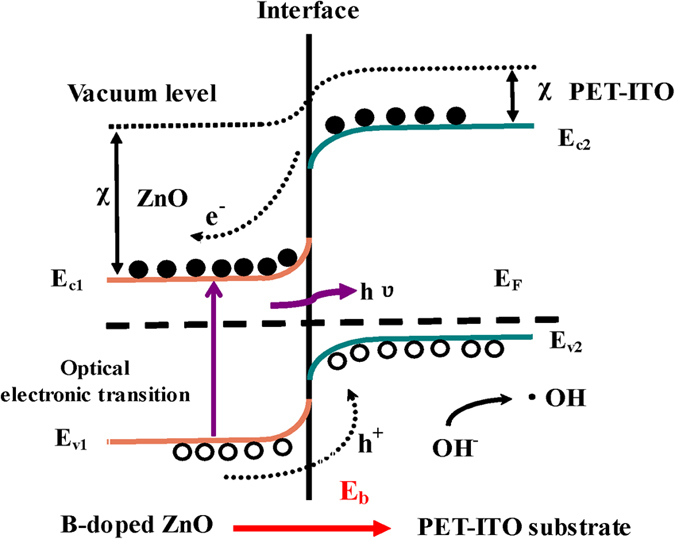
Energy band diagram of the BZO/PET–ITO heterojunction in a thermal equilibrium state. e^−^, electron; h^+^, hole; χ electron affinity; hv, photon energy; E_C_, conduction band; E_V_, valence band; E_F_, Fermi level; ·OH, hydroxyl radical; E_b_, built-in electric field.

The photocatalytic reaction principle of the sheet-sphere-shaped BZO grown on PET–ITO is analyzed by means of energy band theory without considering the interface state (Fig. 11). When the BZO/PET–ITO heterojunction is under UV luminescence with a wavelength of 365 nm (3.397 eV), electrons at the top of the valence band can absorb the photon energy to directly transit to the conduction band. The photo-induced carrier of holes (electrons) is created in the valence band (conduction band) of ZnO and PET–ITO. This principle is similar to the ref. 35 reported by Low *et al*., in which the TiO_2_-based p-n heterojunction photocatalyst be used to CO_2_ reduction. The built-in electric field (E_b_) presented at the interface forces holes (electrons) to transfer from the valence band of n-type BZO (conduction band of PET–ITO) to the valence band of p-type PET–ITO (conduction band of BZO). The effective separation of the photo-production charge carriers increases, which greatly reduces the probability of electron-hole pair recombination and extends the lifetime of photo-generated carriers, result in the activity of ZnO/PET–ITO worked as the photocatalyst is accelerated. Moreover, the light-generated electrons can be captured by the oxidizing substances in the mixed solution to proceed the reduction reaction, so that the O_2_ dissolved in water is transformed into the superoxide anion free radical O_2_^−^, 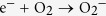. The H_2_O and OH^−^ adsorbed on the surface of the ZnO nanostructures can be oxidized to the hydroxyl radical ·OH by the photo-generated holes acting as a strong oxidizing agent.

The chemical reactions occurring in the above process are shown below.


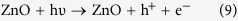



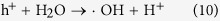



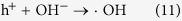


Meanwhile, the ·OH established on the ZnO surface is also a powerful oxidizing agent, with an oxidation potential of 2.8 V (inferior only to fluorine in nature). Therefore, in the course of the photocatalytic reaction of RY 15, the agency with the capability of catalytic decomposition can be classified into the following three kinds: (i) free radical anion, O_2_^−^, where the interaction between the product derived from the uniform fracture of chemical bond when the dye was excited^36^ and the superoxide O_2_^−^ resulted in RY 15 de-colorization; (ii) photo-generated holes, where the chromophoric group was directly degraded by holes; (iii) hydroxyl radical, ·OH, the indirect oxidative damage of chromophore by ·OH. So that, the RY 15 solution was first performed to the de-colorization reaction, and then further oxidized, until it was mineralized to carbon dioxide, water and other inorganic ions. From the above discussion, we know that the ZnO/PET–ITO heterojunction has a particular photocatalytic degradation performance.

According to band theory, the degradation efficiency of B-doped ZnO grown on PET–ITO is higher than the pure ZnO nanostructure. On the one hand, more electron carriers are offered by the boron addition to participate in the redox reaction, making the chromophoric group in the RY 15 is directly oxidized and degraded. Another reason is that after incorporation of boron as a donor impurity into the n-type ZnO, the concentration of donor impurity in the n-region of the p-n junction increases, allowing more electrons to be ionized than in the case of non-doping, and the diffusion movement of electrons from the n-region to the p-region is further increased. Thereby, forming a built-in electric field larger than that of without B-doping, results in further separation of the photo-generated electron-hole pairs and reduction of the recombination probability, increasing the quantity of O^2−^ and ·OH in aqueous solution and eventually achieving the purpose of facilitating the photocatalytic efficiency of the photocatalyst.

## Conclusions

In summary, using Zn(NO_3_)_2_·6H_2_O, C_6_H_12_N_4_, and H_3_BO_3_ as raw materials, sheet-sphere-shaped BZO grown on PET–ITO flexible substrates had been successfully acquired via hydrothermal processing (90 °C, 5 h). The addition of boric acid solution greatly influenced the growth habit of ZnO crystal, and changed the growth rate of each crystal face, which led to the appearance of the lamellar-spherical morphology. The BZO/PET–ITO heterojunction diode exhibited definite rectification characteristics, with several current transport mechanisms present in system; the reason for the reverse leakage current in the unsaturated state was investigated in detail. The photocatalytic properties of ZnO nanostructures were improved by doping with boron, so the degradation rate increased from 20.4% to 41.45%. This was due to the following two reasons: an increasing number of electron carriers were involved in the redox reactions, and the enhancement of the built-in electric field further separated the photo-induced electron-hole pair, so that the content of holes, O^2−^, and ·OH as the strong oxidant were enlarged.

## Additional Information

**How to cite this article**: Wang, W. *et al*. Electrical and photocatalytic properties of boron-doped ZnO nanostructure grown on PET–ITO flexible substrates by hydrothermal method. *Sci. Rep.* **7**, 42615; doi: 10.1038/srep42615 (2017).

**Publisher's note:** Springer Nature remains neutral with regard to jurisdictional claims in published maps and institutional affiliations.

## Figures and Tables

**Table 1:** The kinetic equation and reaction rate coefficient of the photocatalytic experiment.

Photo-catalyst	Kinetic equation	Reaction rate/min^−1^
ZnO nanorods/PET–ITO	In(C_0_/C) = 0.00191t–0.00588	0.00191
BZO sheet-sphere/PET–ITO	In(C_0_/C) = 0.00485t–0.02226	0.00485
